# Stand Competition Determines How Different Tree Species Will Cope with a Warming Climate

**DOI:** 10.1371/journal.pone.0122255

**Published:** 2015-03-31

**Authors:** Laura Fernández-de-Uña, Isabel Cañellas, Guillermo Gea-Izquierdo

**Affiliations:** 1 Forest Research Centre, Instituto Nacional de Investigación y Tecnología Agraria y Alimentaria (INIA-CIFOR), Madrid, Spain; 2 Centre Européen de Recherche et d'Enseignement de Géosciences de l'Environnement (CEREGE), Unité Mixte de Recherche 7330, Centre National pour la Recherche Scientifique/Université Aix-Marseille, Aix-en-Provence, France; Chinese Academy of Sciences, CHINA

## Abstract

Plant-plant interactions influence how forests cope with climate and contribute to modulate species response to future climate scenarios. We analysed the functional relationships between growth, climate and competition for *Pinus sylvestris*, *Quercus pyrenaica* and *Quercus faginea* to investigate how stand competition modifies forest sensitivity to climate and simulated how annual growth rates of these species with different drought tolerance would change throughout the 21st century. Dendroecological data from stands subjected to thinning were modelled using a novel multiplicative nonlinear approach to overcome biases related to the general assumption of a linear relationship between covariates and to better mimic the biological relationships involved. Growth always decreased exponentially with increasing competition, which explained more growth variability than climate in *Q*. *faginea* and *P*. *sylvestris*. The effect of precipitation was asymptotic in all cases, while the relationship between growth and temperature reached an optimum after which growth declined with warmer temperatures. Our growth projections indicate that the less drought-tolerant *P*. *sylvestris* would be more negatively affected by climate change than the studied sub-Mediterranean oaks. *Q*. *faginea* and *P*. *sylvestris* mean growth would decrease under all the climate change scenarios assessed. However, *P*. *sylvestris* growth would decline regardless of the competition level, whereas this decrease would be offset by reduced competition in *Q*. *faginea*. Conversely, *Q*. *pyrenaica* growth would remain similar to current rates, except for the warmest scenario. Our models shed light on the nature of the species-specific interaction between climate and competition and yield important implications for management. Assuming that individual growth is directly related to tree performance, trees under low competition would better withstand the warmer conditions predicted under climate change scenarios but in a variable manner depending on the species. Thinning following an exponential rule may be desirable to ensure long-term conservation of high-density Mediterranean woodlands, particularly in drought-limited sites.

## Introduction

Changes in forest productivity [1], tree phenology [2] and species distribution, both in latitude [3] and altitude [4], have been observed as a result of climate change [5]. Its long-term effects can be, however, modulated by other factors, such as CO_2_ and nitrogen fertilization [5,6] or stand dynamics affected by natural disturbances, forest management, and inter-tree relationships. Competition, both above- and belowground, decreases individual radial growth as a result of reduced resource availability, more negative water potentials and decreased photosynthetic rates and stomatal conductance [7–9]. Consequently, competition affects the variability of the tree growth response to climate [9–12].

Dendrochronological methods provide us with long series of growth data with an annual resolution. These datasets can be used to analyse forests’ response to spatio-temporal changes in climate. However, due to a lack of long-term competition series, there are few studies that have been able to profit from the valuable long time span of this type of data to investigate the effect of the interaction between competition and climate on tree growth. Consequently, most studies addressing the effect of competition on the growth response to climate have analysed this relationship by comparing different competition classes or thinning regimes [10–12], whereas fewer studies explicitly modelled growth as a function of both climate and competition [13,14]. Thus, there is still much need to understand the underlying relationship between competition and climatic variability because these studies have traditionally simplified the growth response to environmental variables and assumed a linear relationship between them. Nonetheless, the physiological response to environmental forcing, and hence the growth response, is not linear [15–18]. Concretely, the functional response to a specific environmental variable has either a sigmoid form, when it increases until reaching a saturation state, such as the response of photosynthetic rates to light [15,19], or a bell-shaped form, when the response to the environmental factor presents an optimum or optimal range, e.g. the effect of nitrogen on growth [15,19]. Because nonlinear approaches can empirically model the biological mechanisms that control the relationship between growth and the interaction between the different environmental variables [15,20], they have greater power to predict growth under past and future climatic conditions [16,20,21].

In the Mediterranean region, drought is a key ecological factor determining plant performance and species distribution [1,22]. Climate change scenarios forecast rising temperatures with stable or even decreasing precipitation, further increasing the frequency and intensity of drought events [23]. Within the region, mountainous areas with colder and more humid climates have served as a refuge for boreal species. These species mingle with Mediterranean taxa increasing overall biodiversity [24]. Consequently, Mediterranean mountainous ecosystems could be particularly vulnerable to species loss under climate change scenarios [3]. The lower belt of the mountains of the central Iberian Peninsula is currently dominated by sub-Mediterranean species such as *Quercus pyrenaica* Willd. and *Quercus faginea* Lam., whereas the higher areas with colder, more continental climates are covered by conifer species such as *Pinus sylvestris* L. and *Pinus nigra* J.F. Arnold [24]. This distribution has been, however, highly influenced by various cycles of deforestation and reforestation in historical times [25]. *P*. *sylvestris* was particularly favoured in reforestation during the 19^th^ century and currently covers 16.2% of the Iberian and Central Mountain Ranges [24,25]. Nevertheless, this Eurosiberian species finds its southwestern distribution limit in the Iberian Peninsula mountain ranges, where its natural populations are highly fragmented [24,25]. Consequently, as a result of climate change, *P*. *sylvestris* is expected to reduce its current distribution range, particularly in altitude, being displaced at low elevations by sub-Mediterranean species such as *Q*. *pyrenaica* and *Q*.*faginea* [24,26,27]. These two species are only found in the transition between the temperate and the Mediterranean zones and together cover 30.3% of the above-mentioned mountain ranges [24,26]. They were traditionally managed as coppices or coppices with standards for firewood extraction, which favoured vegetative regeneration [28–30]. As a result of the abandonment of their traditional use, the stands of these two species have reached high densities with little structural diversity [28–30], which could increase their vulnerability to climate change. *Q*. *faginea* has a higher drought tolerance than *Q*. *pyrenaica*, and, although both species are better adapted to drought than *P*. *sylvestris*, they withstand lower water potentials than Mediterranean evergreen species such as *Quercus ilex* L. [31–34]. Consequently, with climate change, sub-Mediterranean *Quercus* species could be replaced in their lower and southern limits by more drought tolerant taxa [24,26,27].

Given the large area covered by these species in Mediterranean mountain ranges, understanding how *P*. *sylvestris*, *Q*. *pyrenaica* and *Q*. *faginea* stands will respond to changing climatic conditions is essential to assess which management practices would minimize the potentially adverse ecological and socio-economic impacts and ensure their conservation. This study first aims to describe and simulate the relationship between tree growth, climate and competition via a biologically meaningful nonlinear approach, using dendrochronological data and long-term competition series from stands subjected to different thinning regimes. Second, we apply these models to various climate change scenarios to project the future growth trends of these species at different stand competition levels to assess their vulnerability to climate change. Specifically, we were interested to analyse how competition modifies the growth response to climate. We hypothesized that the net effect of climate upon growth would be limited by a competition scale-dependent relationship and that the effect of the interaction between climatic factors and competition on growth would depend on the functional characteristics of the target species, particularly its drought tolerance.

## Materials and Methods

### Study sites

**Fig 1:**
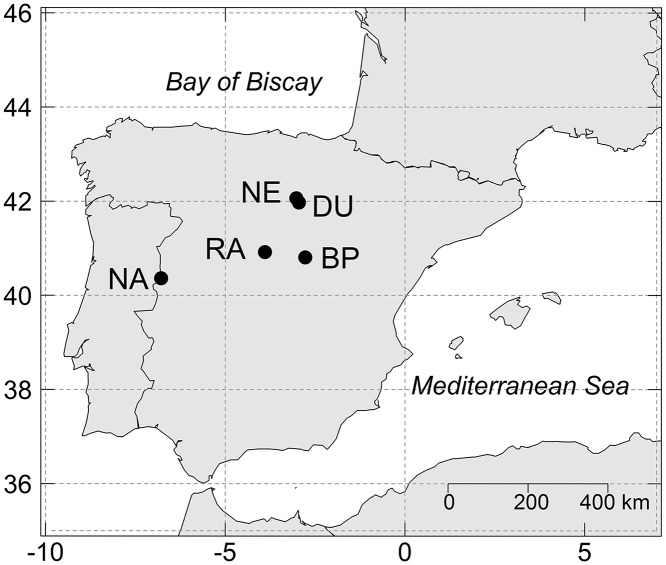
Map of the Iberian Peninsula with the location of the study sites. BP: Barriopedro (*Q*. *faginea*); NA: Navasfrías (*Q*. *pyrenaica*); RA: Rascafría (*Q*. *pyrenaica*); DU: Duruelo (*P*. *sylvestris*); NE: Neila (*P*. *sylvestris*).

We selected different locations for each of the study species within the INIA network of long-term thinning experimental plots: Barriopedro (BP) for *Q*. *faginea* (QUFG); Navasfrías (NA) and Rascafría (RA) for *Q*. *pyrenaica* (QUPY); and Duruelo (DU) and Neila (NE) for *P*. *sylvestris* (PISY; Fig 1). The site characteristics are detailed in Table 1. All plots were located in even-aged, monospecific, naturally regenerated stands representative of the dominant woodlands currently found within the region. *Quercus* spp. stands were traditionally managed as coppice forests. Multiple stems per tree were common at the *Q*. *faginea* site, whereas the trees at the *Q*. *pyrenaica* sites generally had only one stem. At each site, 770-1600-m^2^ plots were marked and randomly either assigned a thinning treatment (light thinning—15–25% plot basal area [BA] reduction—, moderate thinning—35% BA reduction—, or heavy thinning—up to 50% BA reduction) or left unaltered for control purposes, with at least one repetition per treatment. Thinning from below (i.e., thinning that removes the smallest trees in the stand) was performed the year of plot establishment (Table 1) and in approximately 10-year rotation periods. Diameter at breast height (DBH) of all trees was measured in all plots every 4–10 years since plot establishment. Plots were established and are periodically thinned and inventoried with the authorization of the regional governments of Castilla-La Mancha (BP), Castilla y León (NA, DU and NE) and Madrid (RA). No specific permits were required for sampling at these sites, because the study did not involve endangered or protected species nor did it have any potential long-term effects on the sampled trees.

**Table 1:** Site characteristics.

Site	Species	Age	Est. year	N plots	Altit. (masl)	Soil type	Expos.	Slope (%)	Prec. (mm)	Temp. (°C)	Stand type	Treatments	Average DBH (mm)	Plot BA (m^2^/ha)	Density (trees/ha)
BP	*Q*. *faginea*	59	1980	21	860	Haplic calcisol	W	0–35	505	10.5	Xeric	C, L, M, H	88±22–123±28	2.4–19	675–4090
NA	*Q*. *pyrenaica*	64	2004	9	895	Haplic umbrisol	W	0–10	927	10.7	Mesic	C, L, M	188±51–199±49	15–28	430–1100
RA	*Q*. *pyrenaica*	53	1994	8	1400	Haplic umbrisol	SE	30	790	9.4	Mesic	C, L, M, H	133±47–161±39	17–40	765–5490
DU	*P*. *sylvestris*	78	1968	8	1200	Haplic umbrisol	NW	15	860	7.3	Mesic	C, L, H	208±61–288±52	18–56	440–2870
NE	*P*. *sylvestris*	71	1972	9	1340	Haplic umbrisol	NE	15–20	860	6.6	Mesic	C, L, M	242±66–301±67	35–75	350–4600

Age: Average cambial age at breast height in 2014; Est. year: Year of plot establishment (i.e., year in which plots were marked and the first thinning was applied); Altit.: Altitude; C: Control; L: Light thinning; M: Moderate thinning; H: Heavy thinning; Expos.: Exposure; Prec.: Mean annual precipitation; Temp.: Mean annual temperature; DBH: diameter at breast height; Plot BA: Plot basal area. Average diameter is that of the most recent inventory and includes the standard deviation. Average DBH, plot BA and density show the minimum and maximum values per plot for each site.

### Dendrochronological data

At each plot, 20 trees with diameters in the first inventory (i.e., before thinning was applied) above the plot’s average were selected and sampled between 2010 and 2012. In trees with multiple stems (such as the case of control plots at the *Q*. *faginea* site), the largest stem was sampled. Two cores were sampled from each tree at 1.3 m height, with a total of 1100 trees from 55 plots. Cores were air dried, mounted and sanded and measured with a LINTAB measuring table (Rinntech, Heidelberg, Germany) with an accuracy of 0.01 mm. Tree ring series were visually and statistically crossdated with the software TSAP [35], using the statistics Gleichläufigkeit (Glk), t-value and the crossdating index (CDI). Crossdating was finally verified with COFECHA [36].

Ring-width data were transformed into basal area increments (BAI) in mm^2^/year. BAI series were averaged per tree and single tree BAI series were in turn averaged per plot to obtain 55 plot BAI chronologies. Average plot chronologies included only those years for which at least five individual tree series existed. BAI series were used because, with the exception of the first few years of increasing juvenile growth, they minimize the tree-size and age effects while conserving both the high and low frequency signal in the tree ring series [37]. Because prewhitening BAI chronologies to eliminate autocorrelation also removed the short-term growth variations due to changes in competition that we aimed to model, we used raw BAI chronologies as the dependent variable in our models.

### Competition data

Data from the periodic plot inventories were used to build annual chronologies of tree density and basal area (in stems/ha and m^2^/ha, respectively), which were used as proxies of competition at the plot level as they reflect the degree of crowding in the stand [14,38]. Competition series ranged between 9 and 42 years long. We assumed that mortality between inventories occurred gradually in such a way that the annual changes were equal to the difference between inventories divided by the number of years between them. Harvested trees were added to the estimated natural mortality of the year of thinning when this was performed.

### Climate data

Climate data were obtained from the Spanish Meteorological Agency (AEMET), the Peñalara Natural Park Research and Management Centre, Barriopedro site’s meteorological station (in operation from 1980 to 1991), Herrera *et al*. [39] and the CRU TS 3.10 dataset ([40]; accessed 2015 Feb 25 through the KNMI explorer, available http://climexp.knmi.nl/). We considered the climatic data from the weather station located closest to each study site (at 3–19 km away) and at a similar altitude to be the same as in our plots. Missing data were estimated using linear regressions between the data from that reference station and data from the closest weather stations and, when this was not possible, with data from Herrera *et al*. [39] or CRU. Because Neila did not have a station nearby at the same altitude, we used for that site the same precipitation data as for Duruelo, correcting temperature data with a lapse rate of 0.5°C/100 m [41]. Monthly and seasonal precipitation, mean temperatures, mean maximum temperatures and mean minimum temperatures were used for the study. Months were pooled as follows: Annual (January–December), Hydrological year (October of previous year—September of current year), Growing season (April-September), Spring (March-May), Summer (June-August), MJJ (May-July), JJ (June-July), Autumn (September-November) and Winter (December of previous year-February of current year).

### Data analysis

We calculated Kendall rank correlation coefficients between BAI and the various precipitation and temperature variables to narrow down the covariates that were to be tested in the models. We used nonparametric tests because BAI data were not normally distributed. We also explored the data visually to detect non-linear relationships between covariates. The three species differed in the variables triggering a maximum response in growth and, therefore, we calibrated a model per species.

We modelled growth using a nonlinear multiplicative approach [17], which allows modelling the nonlinear relationships between variables and investigating the interactions among them, as well as including the effect of the most strongly limiting factor. Growth was estimated as a function of the maximum potential growth (MG; BAI in mm^2^/year), i.e., the potential tree growth when all the environmental variables are at their optimum, multiplied by functions of tree size, competition and climatic variables (temperature and precipitation) with values enclosed between 0 and 1 [17]. Because age was highly correlated with tree size (Kendall's τ coefficient = 0.55–0.83), this variable was not included in the model. Therefore, the general form of our model was:

BAI=MG·f1(Size)·f2(Competition)·f3(Precipitation)·f4(Temperature)+ε (1)

where *f*_*i*_ *(x)* are unitless functions (modifiers) representing the functional relationships between growth and the different covariates and *ε* is the random error.

Several functions per covariate were compared to choose the function that best fitted the data (Table 2):

**Table 2:** Functions assessed, where *x* may be size, competition, precipitation or temperature variables, and f(x) ~ [0, 1].

Function ID	Function type	Function general expression	Variables
1	Logistic	$f(x)=\frac{1}{1+{\left(\frac{x}{a}\right)}^{b}}$	Size; Precipitation; Temperature
2	Modified Gaussian	$f(x)=e^{-0.5{\left(\frac{x-a}{b}\right)}^{c}}$	Competition; Precipitation; Temperature
3	Exponential	*f(x) = ae* ^*bx*^	Competition
4	Potential	*$f(x)=a^{bx}$f*(*x*) *= a* ^*bx*^	Competition
5	Log-normal	$f(x)=e^{-0.5{\left(\frac{\log\left(\frac{x}{a}\right)}{b}\right)}^{2}}$	Temperature
6	Modified Laplace	$f(x)=e^{{\left(-\frac{\left|x-a\right|}{0.5}\right)}^{b}}$	Temperature

**Fig 2:**
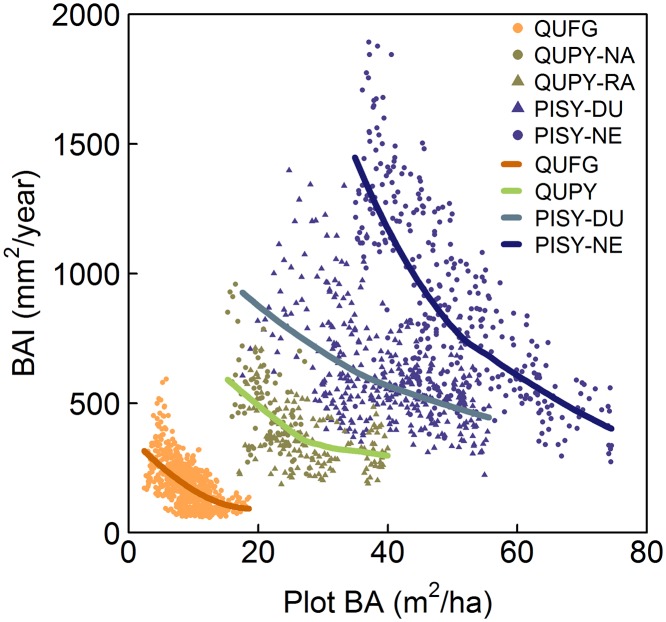
BAI (mm^2^/year) plotted against stand basal area (BA) per site. The relationship between competition and growth follows a negative exponential form in all sites, as shown by locally weighted polynomial regression (loess smoother) lines.

1. *f*_*1*_ *(Size)*: The effect of size (mean annual diameter of the trees forming the chronology in mm) followed a logistic function (function 1 in Table 2), and thus increased up to an asymptote.
2. *f*_*2*_ *(Competition)*: As suggested empirically by our data (Fig 2) and expressed in the literature [12,17], the relationship between BAI and competition followed a decreasing curve. Thus, we compared different formulations to represent this response, including the modified Gaussian, negative exponential and negative potential functions (functions 2 through 4 in Table 2). For PISY, we allowed the parameters in the competition function to vary with site because the competition curves from DU and NE were parallel (Fig 2). This was most likely a result of different site characteristics, such as soil fertility, not explained by our model.
3. *f*_*3*_ *(Precipitation)*: The relationship between growth and precipitation was expected to be represented by an increasing monotonic function with an asymptote [15,16]. Nonetheless, to ensure that growth did not decrease with increasing precipitation after an optimum, we compared the logistic function with a modified Gaussian (functions 1 and 2 in Table 2).
4. *f*_*4*_ *(Temperature)*: We expected temperatures to present an optimum or optimal range at which trees perform best [15,16]. Consequently, we compared logistic, modified Gaussian, log-normal and modified Laplace functions (functions 1, 2, 5 and 6 in Table 2) to cover a variety of shapes reflecting different ecological responses to temperature.

Models were fitted using maximum likelihood. Model parameters, including MG and individual function parameters, were estimated using the global optimization algorithm ‘simulated annealing’ [42]. Various combinations of response functions and covariates preselected on the exploratory analysis were compared to select the covariate and response function that exhibited the strongest relationship with growth for each modifier in Eq 1 (i.e., one for competition, one for precipitation and one for temperature). The best model was selected based on the models’ log-likelihood, Akaike Information Criterion (AIC), adjusted coefficient of determination (R^2^) and root mean square error (RMSE) [43]. A model was considered to have a significantly higher explanatory power than another when the difference in AIC between models (ΔAIC) was equal to or greater than 2 [44]. Because data and residuals were heteroscedastic and presented positive kurtosis, a Gamma probability density function was used to fit ε in the models.

### Growth projections under different climatic and competition scenarios

To project growth at the various studied sites by applying the selected best models, we used climatic scenarios of monthly minimum and maximum temperatures and precipitation of the study sites obtained from the University of Cantabria (http://www.meteo.unican.es/en/projects/estcena; Accessed 2015 Feb 25). Projections used the general circulation model ECHAM5 for the IPCC emission scenarios A1B, A2 and B1 [23]. Since climate predictions were made for 20 x 20 km grids, data were adjusted to the study sites when necessary using the overlapping period 2001–2011, common to both the data used to calibrate the models and the future predictions.

We projected two types of scenarios based on tree size. First, we projected the growth of the studied stands throughout the 21^st^ century, increasing tree diameter based on the BAI predicted for previous years (hereafter dynamic projections). Second, we assessed the changes in annual growth that a given size class would suffer as a result of climate and competition, for which we used the same diameter per size class throughout the simulation period (hereafter constant-diameter projections). We estimated BAI for two different size classes per species: 100 and 150 mm-diameter for QUFG, 150 and 200 mm for QUPY and 200 and 300 mm for PISY. We considered two competition scenarios: heavy thinning (HT) and control (C). For the dynamic projections, in the heavy thinning scenario we presumed that competition would be kept steady at the same level as at present through forestry practices. Because under natural conditions competition rates are expected to increase up to a maximum level at which rates remain constant through self-thinning [45], for the control scenario we assumed that competition levels would increase over time at rates similar to those measured in the most recent years. We estimated for each site a specific asymptote reflecting constant competition rates based on observed trends and maximum competition levels found in National Forest Inventory data. For the constant-diameter projections, we kept competition levels constant at the same levels as in the present for both the control and heavy thinning scenarios. Climate and competition projections were introduced in the selected models to predict growth throughout the 21^st^ century. For a better assessment of the long-term trends, a smoothing spline with a 50% frequency cut-off at 30 years was applied to the simulated growth series.

All analyses were carried out with R version 2.13.1 [46]. For BAI calculations and future growth projection smoothing we used the package “dplR” [47], while models were fitted using the package “likelihood” [48].

## Results

Descriptive statistics of the studied 55 tree ring chronologies can be found in S1 Table. QUFG had the slowest growth, followed by QUPY. Growth rates were lower at the driest sampled site for each species (S1 Table and Table 1). Trees were relatively young, with chronologies ranging between 54 and 86 years in length (S1 Table).

### Growth model

**Fig 3:**
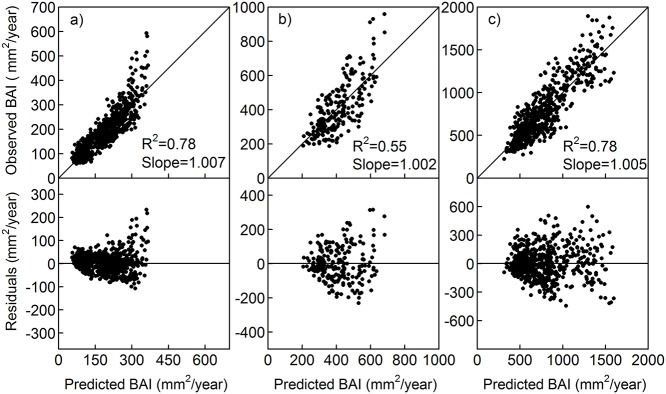
Observed vs. Predicted BAI and Residual plots for the models for (a) *Quercus faginea*, (b) *Quercus pyrenaica* and (c) *Pinus sylvestris*.

**Fig 4:**
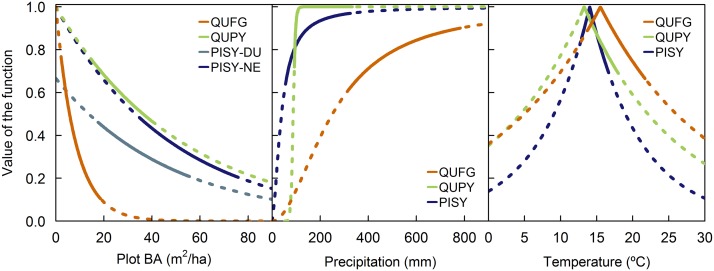
Comparison of the different functional relationships between growth and the various environmental variables as fitted in the models for each of the studied species. Solid lines represent the range for which the models were calibrated, whereas dotted lines represent extrapolations of the models. For *Q*. *faginea*, Precipitation = P_Hyd_ and Temperature = Tmax_Spr_; for *Q*. *pyrenaica*, Precipitation = P_Spr_ and Temperature = Tmax_Spr_; and for *P*. *sylvestris*, Precipitation = P_MJJ_ and Temperature = Tmax_Hyd_. Note that to ease the comparison among species of the different functional relationships, growth is shown on a relative scale for each species, with 1 representing the maximum growth of the species.

The variance explained by the models (R^2^) ranged from 55% for QUPY to 78% for QUFG and PISY (Table 3 and Fig 3). The effect of size on growth was stronger on QUPY and PISY, whereas most of the growth variability of QUFG was explained by competition. For QUFG and PISY, the effect of competition on growth was stronger than that of climatic variables, whereas QUPY growth was more influenced by temperature than by competition (Table 3). Of the two climatic variables assessed, temperature had a stronger influence on both *Quercus* species growth than precipitation, whereas PISY responded more strongly to the latter (Table 3). For all species, plot BA better reflected the effect of competition changes in the stand than tree density. The relationship between growth and competition followed a negative exponential function in all cases (function 3 in Table 2; Figs 2 and 4). QUPY and PISY responded similarly to competition, whereas QUFG suffered stronger decreases in growth with increasing competition (Table 4 and Fig 4).

**Table 3:** Effect of adding one more environmental variable to the nonlinear model.

	QUFG					QUPY					PISY				
Model	LL	ΔAIC	R^2^	RMSE	N par	LL	ΔAIC	R^2^	RMSE	N par	LL	ΔAIC	R^2^	RMSE	N par
Size	-3830.6	925.4	0.11	84.1	4	-1390.4	95.7	0.24	129.2	4	-4783.5	719.0	0.39	250.3	4
Size·Prec·Temp	-3787.6	847.4	0.20	79.7	8	-1354.9	33.2	0.45	110.1	8	-4593.1	346.3	0.63	195.0	8
Size·Comp	-3459.1	184.5	0.70	48.6	5	-1375.7	68.3	0.35	119.4	5	-4524.0	203.9	0.70	175.3	6
Size·Comp·Prec	-3422.3	114.9	0.73	46.2	7	-1356.2	33.6	0.46	109.4	7	-4454.8	69.7	0.76	159.3	8
Size·Comp·Temp	-3406.0	82.4	0.75	45.0	7	-1342.5	6.2	0.53	102.3	7	-4486.3	132.6	0.73	166.6	8
Size·Comp·Prec·Temp	-3362.9	0	0.78	42.1	9	-1337.2	0	0.55	100.1	9	-4417.9	0	0.78	152.7	10

Comp: basal area for all species; Prec: P_Hyd_ for *Q*. *faginea*, P_Spr_ for *Q*. *pyrenaica* and P_MJJ_ for *P*. *sylvestris*; Temp: Tmax_Spr_ for *Q*. *faginea* and *Q*. *pyrenaica* and Tmax_Hyd_ for *P*. *sylvestris*; LL: Log-likelihood; ΔAIC: difference between the AIC of the model being considered and the AIC of the complete model (Size·Comp·Prec·Temp); R^2^: Adjusted coefficient of determination; RMSE: Root mean square error; N par: Number of parameters in the model.

**Table 4:** Values and support intervals of the parameters for the different models.

Parameter	QUFG	QUPY	PISY	
			DU	NE
MG	1055.2 (1044.1, 1065.7)	1965.9 (1908.9, 2006.5)	5546.8 (5491.3, 5602.3)	
Size_a	83.8 (83.8, 83.8)	227.5 (220.7, 234.3)	205.7 (203.6, 207.8)	
Size_b	-5.26 (-5.74, -4.92)	-1.48 (-1.66, -1.32)	-2.09 (-2.27, -1.95)	
Comp_a	1	1	0.6660 (0.6526, 0.6726)	1
Comp_b	-0.1217 (-0.1243, -0.1181)	-0.0191 (-0.0206, -0.0178)	-0.0209 (-0.0214, -0.0203)	
Prec_a	221.0 (211.3, 229.9)	87.7 (85.0, 89.5)	39.0 (36.3, 41.4)	
Prec_b	-2.13 (-2.31, -1.99)	-20.27 (-32.16, 12.96)	-1.63 (-1.79, -1.52)	
Temp_a	16.1 (16.1, 16.4)	13.3 (13.0, 13.5)	14.0 (14.0, 14.0)	
Temp_b	0.0312 (0.0274, 0.0337)	0.0393 (0.0334, 0.0482)	0.0701 (0.0638, 0.0745)	

MG: maximum growth (mm^2^); Size_a and Size_b: parameters of the size function (logistic), which represent the half saturation point and the scale of the function, respectively; Comp_a and Comp_b: parameters of the competition function (negative exponential), which represent the intercept and the shape of the function, respectively; Prec_a and Prec_b: parameters of the precipitation function (logistic); Temp_a and Temp_b: parameters of the temperature function (modified Laplace), which represent the optimum temperature and the scale of the function, respectively.

The relationship between growth and the climatic variables also followed the same functional form for the three species, although the precipitation and temperature variables triggering this response were species-specific. The logistic function best captured the relationship between precipitation and growth (function 1 in Table 2), indicating that the growth response reached an asymptote with increasing moisture availability, whereas the modified Laplace (function 6, a distribution with a single maximum located at a sharply pointed peak) worked best for temperatures (Fig 4). This function suggests that growth increased exponentially up to an optimum, after which growth decreased exponentially with increasing temperatures. The characteristics of this growth response to temperature were similar for both *Quercus* species, whereas changes in growth with temperature were steeper in PISY. For the analysis of the climatic variables affecting growth, only the final climate variables selected for the models are given due to the large number of variables tested. QUFG responded more strongly to precipitation of the hydrological year (P_Hyd_) and spring maximum temperatures (Tmax_Spr_), whereas QUPY responded to spring precipitation (P_Spr_) and maximum temperatures (Tmax_Spr_). PISY growth was mostly related to May-July precipitation (P_MJJ_) and maximum temperatures of the hydrological year (Tmax_Hyd_).

### Growth projections under climate change scenarios

**Fig 5:**
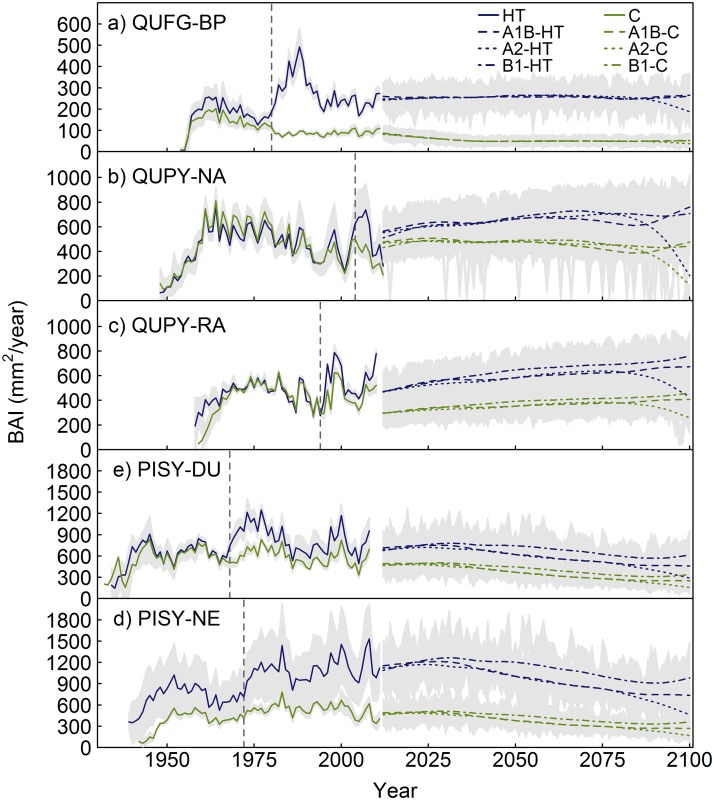
Mean chronologies and dynamic growth projections under different competition (Control, C, and Heavy thinning, HT) and climate change (A1B, A2 and B1) scenarios for the different study sites. (a) Barriopedro (*Q*. *faginea*); (b) Navasfrías (*Q*. *pyrenaica*); (c) Rascafría (*Q*. *pyrenaica*); (d) Duruelo (*P*. *sylvestris*); and (e) Neila (*P*. *sylvestris*). Grey dashed vertical lines indicate the time when plots were established and thus, the years from which data were used to calibrate the models, which do not include increasing juvenile growth. Shading represents the confidence intervals, calculated as the mean ± standard deviation.

**Fig 6:**
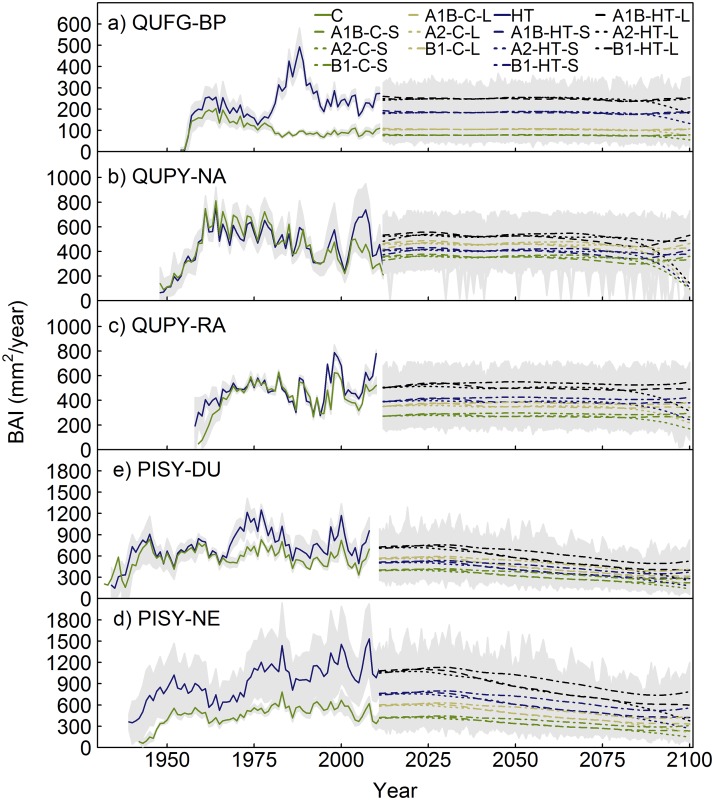
Mean chronologies and constant-diameter growth projections for the various size classes under different competition and climate change scenarios for the different study sites. (a) Barriopedro (*Q*. *faginea*); (b) Navasfrías (*Q*. *pyrenaica*); (c) Rascafría (*Q*. *pyrenaica*); (d) Duruelo (*P*. *sylvestris*); and (e) Neila (*P*. *sylvestris*). Competition scenarios: Control (C) and Heavy thinning (HT). Climate scenarios: A1B, A2 and B1. Size classes: S (100 mm for QUFG, 150 mm for QUPY and 200 mm for PISY) and L (150 mm for QUFG, 200 mm for QUPY and 300 mm for PISY). Shading represents the confidence intervals, calculated as the mean ± standard deviation.

According to the dynamic projections made by our models for the study sites, the forecasted climate change scenarios would affect PISY more negatively than the oaks studied. QUFG growth would experience a decrease in control plots under all climatic scenarios, whereas it would remain constant under the heavy thinning scenario, except for the A2 scenario, which forecasts higher temperature increases [23] (Fig 5). PISY growth would significantly decrease under all size, climatic and competition scenarios assessed at both study sites (Figs 5 and 6). Conversely, QUPY growth would increase during the 21^st^ century under the A1B and B1 scenarios in RA, the QUPY site located at a higher, colder location, whereas in NA this increase would only occur under reduced competition conditions, with stable or slightly decreasing growth trends in control plots (Fig 5). Nonetheless, the constant-diameter projections indicate that these increasing growth trends are due to the positive effect of increasing tree size and not a response to future climate, because they predict that both QUPY and QUFG tree productivity would remain constant or slightly decrease during the 21^st^ century for all size-classes assessed (Fig 6). Under the A2 scenario, however, the models predict a decrease in growth under both competition levels for all sites (Figs 5 and 6).

## Discussion

### Species-specific nonlinear interaction between competition and climate

Our nonlinear model succeeded in capturing the functional response of growth to climate, as well as the nonlinear interaction between climatic variables and stand competition. Precipitation had a positive effect on growth, as expected in water-limited Mediterranean ecosystems [1,10,49,50]. However, this positive effect is nonlinear and ultimately saturates and reaches an asymptote [15,16]. This indicates that in the study areas moisture availability constrains growth up to a species-specific threshold, after which precipitation never reaches high enough levels to limit growth [51]. In contrast, the response to temperature was positive up to an optimum, after which tree growth is limited by increasing temperatures [15,16]. This is consistent with the response of photosynthetic rates to temperature, which also shows an optimum as a result of Rubisco inactivity at low temperatures and stomatal closure with increasing water stress at high temperatures [52–54].

The growth response of each species to climate depends on the species’ ecological requirements, particularly its drought tolerance. According to our model, the growth response to temperature was similar in both *Quercus* species, both in terms of the shape of the function and the strength of the effect, which was more intense than that of precipitation. In contrast, the effect of precipitation was stronger than that of temperature on *P*. *sylvestris*, although its response function to temperature showed that this species has higher sensitivity to temperature changes than do the other two species. This species has its southernmost and, thus, dry distribution limit in the Iberian Peninsula and is less drought tolerant than the studied *Quercus* spp. [27,32], which would explain its stronger dependence on precipitation. The nonlinear response to climatic variables and the species-specific precipitation and temperature thresholds identified by our models improve our understanding of how species will respond under climate change. The assessment of the functional relationships between growth and climatic forcing can therefore improve the reliability of models using long-term dendroecological data. Hence, nonlinear approaches are able to overcome modelling shortcomings resulting from assumptions of linearity such as the ‘divergence problem’ observed in the temperature-tree growth relationship [55,56].

In spite of the substantial influence of climate on growth, the effect of competition was, however, dominant over that of climate for *Q*. *faginea* and *P*. *sylvestris*, as shown for other species [11,49,57]. The functional relationship between growth and competition followed a negative exponential function in all species. However, the responses of *Q*. *pyrenaica* and *P*. *sylvestris* to competition were similar in amplitude, while *Q*. *faginea* suffered stronger growth constraints with increasing competition. *Q*. *faginea* trees presented multiple stems which shared the same root system, and this characteristic caused high competition levels. After thinning, only one stem per tree remained on low competition plots. These single-stem trees most likely profited from larger root systems, which together with the more xeric conditions of this site, could have amplified the effect of competition on this species compared with the other two. This reflects the strong interaction between competition and climatic stress, which our model was able to capture thanks to its multiplicative nature. A proper characterization of this interaction is crucial to understand the combined effect of these two abiotic factors on the response of forests to climate change. Consequently, assuming that growth is a proxy for species performance and, therefore, that reduced growth rates imply enhanced vulnerability, the sustainability of these stands will depend on the species-specific nonlinear interaction between competition intensity and long-term climate forcing, with individuals subject to high competition levels being less likely to survive enhanced xericity [11,49,58,59].

### Growth projections under different climate and competition scenarios. Implications for future management

Most studies on growth projections under climate change scenarios have neglected the importance of competition [60,61], despite the evidence indicating its role in altering tree growth response to climate [10–12,49] and, thus, the need to modify growth projections under future climate scenarios [13]. Our models predicted a reduction of tree growth in stands with high competition levels for *Quercus faginea* and *Pinus sylvestris*, whereas this reduction would be minimized under low competition levels. Multiple studies have recorded the negative effect of high temperatures and drought on tree physiology and growth [62,63], as well as the positive effect of reduced competition for resources on tree performance, particularly under xeric conditions [7–9,50]. Therefore, the predicted decreasing future growth trends and highly reduced growth rates could forecast enhanced tree mortality rates [59,64], particularly under high competition levels. Several studies have already observed an increase in mortality, consistent with self-thinning dynamics, in certain species of the Mediterranean region, including *P*. *sylvestris*, as a result of increased stand competition, rising temperatures and drought episodes [57,65,66]. Increased mortality, together with reduced regeneration [67,68] and decreased growth, could lead to the decline of these stands and their substitution by better adapted species, as predicted by species distribution models [24,26]. Our models suggest that *P*. *sylvestris* growth would be more negatively affected by climate change than that of the two more drought-tolerant sub-Mediterranean *Quercus* spp. studied. This could forecast a displacement in altitude of *Pinus sylvestris* in favour of *Quercus pyrenaica*, for which our models predicted an increase in growth for the site located at a higher elevation, which is consistent with species distribution simulations in the literature [24,26]. Nonetheless, under the warmest climate scenario, *Q*. *pyrenaica* growth would also decline at both sites, indicating that, due to the nonlinear nature of the temperature-growth relationship, the forecasted temperature increase may become limiting for this species growth too [16].

Nevertheless, the mean growth rates predicted under decreased competition for both *Quercus* species indicate that applying competition reductions similar to those assessed with our models could mitigate the potential negative effects of climate change upon growth for trees suffering from stand densification. In contrast, *P*. *sylvestris* would suffer a reduction in growth even under the low-competition scenario, indicating high vulnerability to increasing temperatures. Therefore, heavier thinning intensities than those applied today may be necessary to maintain this species’ tree growth. Because the negative effect of competition declines exponentially with decreasing competition, species-specific thresholds below which growth could be optimised without substantially reducing stand density must be identified, as intense thinning may have detrimental effects on stand productivity and sustainability [28,69]. These thresholds could also be site-dependent, particularly at the edges of each species distribution, where competitive stress may be strengthened due to the nonlinear interaction between competition and climate.

Despite their value for adaptive management, growth projections must be assessed with caution, because there are many inherent uncertainties associated with the extrapolation of models outside their calibration range. One of their main limitations is the impossibility of incorporating potential species acclimation to changing climatic conditions. The nonlinear nature of the models can, however, partially offset this uncertainty because they most likely capture the functional growth response to environmental variability. Moreover, we did not cover the whole climatic range of the studied species. Therefore, these models could still be improved by calibrating them with data from a broader climatic gradient [16] and, thus, extend their applicability to the entire distribution range of each of the studied species. This can be particularly relevant for *Q*. *faginea*, for which we only had one site and for which, therefore, our ability to assess climatic variability was most likely more reduced than that for the other two species. Nonetheless, as long series of competition levels are not readily available, our multispecies approach using stand competition series from long-term experimental plots is particularly valuable.

## Conclusions

Our nonlinear models highlighted the species-specific nature of the growth response to climate and its interaction with competition. These models overcome several limitations of classic linear approaches. Moreover, they shed light on factors that contribute to better understanding of instabilities in the growth response to climate and could be used to detect climatic thresholds of species performance. As a result of this nonlinear interaction, trees under low competition will better withstand the warmer conditions predicted under climate change scenarios, particularly for the two *Quercus* species studied. Competition will most likely be naturally maintained at sustainable levels through self-thinning [45]. However, this process can be accelerated to reduce the vulnerability of the remaining trees to drought through thinning. In light of our results, plot basal area reductions as intense as or even heavier than the ones currently applied may be necessary to prevent stand growth stagnation, particularly in drought-limited sites. However, the assessment of the species-specific competition thresholds should follow an exponential rule, as shown by our models. Although thinning is already commonly applied in *P*. *sylvestris* stands, it still needs to be further developed and implemented for the studied *Quercus* spp. Because proactive approaches are more likely to avoid or reduce damage than reactive ones as they enhance the stand’s resilience, it is important to adapt management in time to prevent possible forest decline and to ensure long-term conservation of high-density Mediterranean woodlands.

## Supporting Information

S1 Table Characteristics of the complete tree ring chronologies averaged per treatment. C: Control; L: Light thinning; M: Moderate thinning; H: Heavy thinning; RW: Ring-Width; Rbar: Interseries correlation; EPS: Expressed Population Signal; MS: Mean sensitivity, AR1: Mean autocorrelation. N trees indicates the total number of trees used to build the chronologies and N cores the number of cores used, which, therefore, does not include those that were discarded.
(DOCX)
