# Correction: Stand Competition Determines How Different Tree Species Will Cope with a Warming Climate

**DOI:** 10.1371/journal.pone.0137932

**Published:** 2015-09-03

**Authors:** The PLOS ONE Staff

There are errors in Equation 1, Table 2 and Table 4 that were introduced during the typesetting process. The publisher apologizes for these errors. Please view the complete, correct Equation 1 here:

$$BAI=MG\cdot f_{1}(Size)\cdot f_{2}(Competition)\cdot f_{3}(Precipitation)\cdot f_{4}(Temperature)+\varepsilon$$

Please see the corrected Table 2 and Table 4 here.

**Table 2:** Functions assessed, where x may be size, competition, precipitation or temperature variables, and f(x) ~ [0, 1].

Function ID	Function type	Function general expression	Variables
1	Logistic	$f(x)=\frac{1}{1+{\left(\frac{x}{a}\right)}^{b}}$	Size; Precipitation; Temperature
2	Modified Gaussian	$f(x)=e^{-0.5{\left(\frac{x-a}{b}\right)}^{c}}$	Competition; Precipitation; Temperature
3	Exponential	*f*(*x*) = *ae* ^*bx*^	Competition
4	Potential	*f*(*x*) = *a* ^*bx*^	Competition
5	Log-normal	$f(x)=e^{-0.5{\left(\frac{\log\left(\frac{x}{a}\right)}{b}\right)}^{2}}$	Temperature
6	Modified Laplace	$f(x)=e^{{\left(-\frac{\left|x-a\right|}{0.5}\right)}^{b}}$	Temperature

**Table 4:** Values and support intervals of the parameters for the different models.

Parameter	QUFG	QUPY	PISY	PISY
			DU	NE
MG	1055.2(1044.1, 1065.7)	1965.9(1908.9, 2006.5)	5546.8(5491.3, 5602.3)	5546.8(5491.3, 5602.3)
Size_a	83.8(83.8, 83.8)	227.5(220.7, 234.3)	205.7(203.6, 207.8)	205.7(203.6, 207.8)
Size_b	-5.26(-5.74, -4.92)	-1.48(-1.66, -1.32)	-2.09(-2.27, -1.95)	-2.09(-2.27, -1.95)
Comp_a	1	1	0.6660(0.6526, 0.6726)	1
Comp_b	-0.1217(-0.1243, -0.1181)	-0.0191(-0.0206, -0.0178)	-0.0209(-0.0214, -0.0203)	-0.0209(-0.0214, -0.0203)
Prec_a	221.0(211.3, 229.9)	87.7(85.0, 89.5)	39.0(36.3, 41.4)	39.0(36.3, 41.4)
Prec_b	-2.13(-2.31, -1.99)	-20.27(-32.16, 12.96)	-1.63(-1.79, -1.52)	-1.63(-1.79, -1.52)
Temp_a	16.1(16.1, 16.4)	13.3(13.0, 13.5)	14.0(14.0, 14.0)	14.0(14.0, 14.0)
Temp_b	0.0312(0.0274, 0.0337)	0.0393(0.0334, 0.0482)	0.0701(0.0638, 0.0745)	0.0701(0.0638, 0.0745)
