# Supplementary material for: Stand Competition Determines How Different Tree Species Will Cope with a Warming Climate
Source: PLoS One. 2015 Mar 31;10(3):e0122255. doi: 10.1371/journal.pone.0122255 (PMC4380403; doi:10.1371/journal.pone.0122255)
Supplement: S1 Table — C: Control; L: Light thinning; M: Moderate thinning; H: Heavy thinning; RW: Ring-Width; Rbar: Interseries correlation; EPS: Expressed Population Signal; MS: Mean sensitivity, AR1: Mean autocorrelation. N trees indicates the total number of trees used to build the chronologies and N cores the number of cores used, which, therefore, does not include those that were discarded. (DOCX) [file pone.0122255.s001.docx]

**Table S1.** **Characteristics of the complete tree ring chronologies averaged per treatment.**

| **Site** | **Treatment** | **N**  **chron.** | **N trees**  **[N cores]** | **Mean**  **length**  **(years)** | **RW (mm)** | | **Rbar** | **EPS** | **MS** | **AR1** |
| --- | --- | --- | --- | --- | --- | --- | --- | --- | --- | --- |
|  |  |  |  |  | **Mean** | **Std. Dev** |  |  |  |  |
| BP | C | 4 | 84[163] | 58 | 0.68 | 0.53 | 0.591 | 0.993 | 0.210 | 0.808 |
|  | L | 5 | 102[198] | 57 | 0.74 | 0.45 | 0.656 | 0.985 | 0.218 | 0.737 |
|  | M | 6 | 120[240] | 58 | 0.89 | 0.51 | 0.778 | 0.981 | 0.222 | 0.726 |
|  | H | 6 | 120[240] | 58 | 0.96 | 0.49 | 0.753 | 0.980 | 0.202 | 0.747 |
| NA | C | 3 | 60[120] | 68 | 1.45 | 0.85 | 0.633 | 0.986 | 0.256 | 0.791 |
|  | L | 3 | 58[116] | 71 | 1.47 | 0.83 | 0.672 | 0.988 | 0.238 | 0.790 |
|  | M | 3 | 60[119] | 69 | 1.43 | 0.74 | 0.710 | 0.984 | 0.249 | 0.743 |
| RA | C | 2 | 40[79] | 54 | 1.34 | 0.83 | 0.486 | 0.988 | 0.237 | 0.814 |
|  | L | 2 | 40 [78] | 54 | 1.30 | 0.74 | 0.527 | 0.987 | 0.227 | 0.811 |
|  | M | 2 | 40[80] | 54 | 1.35 | 0.67 | 0.584 | 0.984 | 0.225 | 0.752 |
|  | H | 2 | 40[79] | 61 | 1.52 | 0.77 | 0.588 | 0.984 | 0.224 | 0.753 |
| DU | C | 3 | 62[124] | 86 | 1.26 | 0.69 | 0.701 | 0.979 | 0.229 | 0.758 |
|  | L | 2 | 40[80] | 82 | 1.35 | 0.75 | 0.718 | 0.986 | 0.224 | 0.777 |
|  | H | 3 | 60[118] | 79 | 1.45 | 0.64 | 0.711 | 0.977 | 0.217 | 0.765 |
| NE | C | 3 | 58[117] | 72 | 1.49 | 0.78 | 0.639 | 0.983 | 0.194 | 0.811 |
|  | L | 3 | 60[120] | 80 | 1.78 | 0.74 | 0.606 | 0.971 | 0.175 | 0.807 |
|  | M | 3 | 60[120] | 80 | 1.86 | 0.77 | 0.655 | 0.978 | 0.181 | 0.803 |

C: Control; L: Light thinning; M: Moderate thinning; H: Heavy thinning; RW: Ring-Width; Rbar: Interseries correlation; EPS: Expressed Population Signal; MS: Mean sensitivity, AR1: Mean autocorrelation. N trees indicates de total number of trees used to build the chronologies and N cores the number of cores used which, therefore, does not include those that were discarded.
